# Editorial: Innovative approaches in glioma therapy: exploring new therapeutic frontiers, volume II

**DOI:** 10.3389/fnmol.2026.1891346

**Published:** 2026-06-19

**Authors:** Pilar Marcos, Arturo Mangas, Rafael Coveñas

**Affiliations:** 1 Human Neuroanatomy Laboratory, Faculty of Medicine, Health Research Institute of Castilla-La Mancha (IDISCAM), Instituto de Biomedicina de Castilla-La Mancha (IB), University of Castilla-La Mancha, Albacete, Spain; 2 Laboratory of Neuroanatomy of the Peptidergic Systems, Institute of Neurosciences of Castilla and León (INCYL), University of Salamanca, Salamanca, Spain; 3 Group GIR USAL: BMD (Bases Moleculares del Desarrollo), University of Salamanca, Salamanca, Spain

**Keywords:** glioma, molecular approaches, patient-based studies, revision, therapies

The second volume of the Research Topic entitled “*Innovative approaches in glioma therapy: exploring new therapeutic frontiers*,” together with the first volume (Marcos et al.), shows the large number of different strategies that exist to combat glioma. In the first volume, molecular targets (ADORA1, Fyn, metformin, ion channels, and clusterin), prognostic assessment (lysine crotonylation-related long non-coding RNAs), anti-glioma therapies (intraoperative radiotherapy, the combination of photodynamic, and tumor-treating fields therapies) and studies focused on pleomorphic xanthoastrocytoma and the involvement of the gut microbiota in glioma were published (Marcos et al.). Building on this, this second Research Topic expands our understanding of various aspects of glioma, ranging from original articles and patient studies to review articles that compile some of the available information on glioma from a multidisciplinary perspective. Both volumes considerably increase our knowledge on glioma from different approaches.

Most of the preclinical models used to study the effectiveness of molecular leads on two-dimensional cell cultures report data on the toxicity exerted against glioblastoma, but they do not provide information about the mechanisms of action of the therapeutic drugs; however, three-dimensional cultures better simulate *in vivo* tumor conditions (Joshi et al.). Using a rotary cell culture technique to culture three-dimensional cancer spheroids of the T98G glioblastoma cell line, the group headed by Dr. Shukla (Pharmacy Sciences Department, School of Pharmacy and Health Professions, Creighton University, Omaha, United States) demonstrated that the specific histone deacetylase (HDAC) inhibitor tubacin exerted a more potent anti-proliferative effect in live-dead and cytotoxicity assays (lower IC_50_ values in two- and three-dimensional cultures and greater toxicity in live-dead imaging), but this inhibitor failed to decrease long-term resistance or migration (Joshi et al.). Vorinostat (a non-specific inhibitor) promoted the migration of these cells at its IC_50_ concentration in two-dimensional cultures, whereas, compared to untreated controls, none of the inhibitors studied significantly decreased long-term resistance (Joshi et al.). The authors concluded that more potent and selective HDAC inhibitors or compounds directed against complementary pathways should be developed in the future for monotherapy in glioblastoma. This study also shows that functional assays, the three-dimensional spheroid-ECM embedded model, and electric cell impedance sensing (ECIS)-based migration assessment are useful procedures for searching for new HDAC inhibitors against glioblastoma, allowing a more consistent evaluation of cell viability, migration/invasion, and resistance phenotypes; this is crucial before performing preclinical *in vivo* studies or translational clinical trials. This procedure will serve not only to study cytotoxic effects but also for functional studies focused on the aggressiveness of glioblastoma.

Dr. Zhao and colleagues (Department of Neurosurgery, The Second Hospital of Hebei Medical University, Shijiazhuang, China) are studying the behavior of various lncRNAs, which are RNAs shorter than 200 nucleotides that have limited or no ability to encode proteins (Yan et al.). These lncRNAs are involved in numerous cancer-related cellular functions, including gliomas, and can be used as biomarkers to help predict patient prognosis. The researchers have focused on ZFHX4-AS1 to study its role in cell proliferation, invasion, and metastasis in glioma. Their studies, which combine bioinformatic approaches, cell cultures, tissue analysis, cell transfection, western blot and RT-PCR, wound healing, xenograft models in nude mice, fluorescence *in situ* hybridization, and co-immunoprecipitation assay, have confirmed previous findings demonstrating that this lncRNA interacts with other genes and promotes the expression of SOX2, accelerating glioma progression. ZFHX4-AS1 promotes glioma cell proliferation *in vitro* and tumor growth *in vivo*, as well as glioma cell invasion and migration. They have also found that glioma cells express ZFHX4-AS1 in both the nucleus and cytoplasm. In addition, the nearby gene *ZFHX4* is highly expressed in gliomas and promotes the growth of glioma cells *in vivo* and the invasion and migration of glioma cells. These results confirm that ZFHX4-AS1 modulates the proliferation, invasion, and migration of glioma cells via its target gene *ZFHX4*. Moreover, ZFHX4 binds to SOX2 and promotes its expression, thereby contributing to the malignant biological behavior of gliomas. ZFHX4-AS1 initiates an oncogenic signal that is amplified by the ZFHX4/SOX2 loop. This combination ultimately converges on the activation of the JAK-STAT pathway. This detailed ZFHX4-AS1/ZFHX4/SOX2/JAK-STAT axis represents a promising set of therapeutic targets for glioma treatment, suggesting for example the use of inhibitors against the ZFHX4-SOX2 interaction (Yan et al.).

The methods used to administer treatments for glioma are still being studied to identify more effective strategies. In this regard, Dr. McDaid and colleagues (Department of Neurosurgery, Endeavor Health, Evanston, IL, United States) have tested the efficacy of administering chemotherapy via an intracerebral osmotic pump in a rat model of glioblastoma (McDaid et al.). It has long been known that the blood-brain barrier limits the effectiveness of chemotherapeutic agents administered systemically; therefore, direct administration at the tumor site may improve their effectiveness. But even under these conditions, when the drug is administered during surgery in the form of carmustine/bis(chloroethyl)nitrosourea (BCNU) wafers, success is limited by the need for wafer solubilization and by the drug's distribution over short distances for a brief period. The authors evaluated the efficacy of administering chemotherapy (two alkylating agents) directly into the tumor cavity in a rat glioma model using convection enhanced delivery (CED) and an implanted microfluidic osmotic pump, whose design is original (McDaid et al.). This methodology, in which the pump contains a high concentration of ferumoxytol [a superparamagnetic iron oxide nanoparticle (SPION)], has proven to be more effective in drug delivery, particularly in terms of tumor recurrence. The combination of MRI perfusion tracking and histology indicates release of SPIONs from the pump at 2 days, and significant perfusion was still observed at 35 days after resection and pump placement. The authors propose that BCNU may be an effective choice for CED-driven, locally delivered chemotherapy in glioblastoma. To translate these findings into clinical practice for the benefit of patients, they suggest developing biodegradable pumps for permanent implantation, which would require the involvement of engineers, neuro-oncologists, neurosurgeons, and others. Dr. McDaid and his colleagues are already working on some prototypes with engineers' partners, which holds promise for the effective administration of treatments (McDaid et al.).

Dr. Ribeiro and coworkers (Department of Internal Medicine, Medical School, Western São Paulo University, São Paulo, Brazil) have reviewed the therapeutic potential of erythropoietin-producing hepatocellular (Eph) signaling in tumors of the central nervous system (Ribeiro et al.). This group reviewed preclinical data and clinical cohorts to evaluate functional outcomes in neoplasms of this system. Ephrin ligands and Eph receptor tyrosine kinases regulate cell proliferation, adhesion, and migration, as well as vascular processes. In glioma and, especially in glioblastoma, an increased invasiveness and higher tumor grade have been associated with EphA2/EphA3 receptor overexpression, whereas the ephrin-A1 and ephrin-A5 ligands exert glioma-suppressive effects by promoting receptor internalization/degradation and inhibiting tumor cell proliferation and migration (Ribeiro et al.). Increased migratory capacity and angiogenesis have been related to a higher expression of EphB1 and EphA4 receptors in medulloblastoma, whereas an aberrant activation of EphA2/EphB1 receptors promotes tumor cell proliferation via the ERBB3 (HER3) and mechanistic target of rapamycin (mTOR) signaling pathways in meningiomas (Ribeiro et al.). Emerging therapeutic strategies (e.g., selective kinase inhibitors, ephrin-based immunomodulators, and ligand-targeted cytotoxins) have exerted anti-cancer effects in preclinical studies, suggesting that targeting the Eph/ephrin system has enormous translational potential. Accordingly, this system is a promising therapeutic target to fight central nervous system tumors.

Dr. Valvi and colleagues (Department of Pediatric and Adolescent Oncology and Hematology, Perth Children's Hospital, Perth, WA, Australia) have studied high-grade gliomas with isocitrate dehydrogenase (IDH) mutations, with a particular focus on adolescent and young adult patients (Valvi et al.). The authors focus on this population, as it is the least studied of all age groups. These gliomas are of particular interest because they exhibit distinct clinical, pathological, and molecular characteristics, including a much more favorable prognosis and response to treatment than their wild-type counterparts. In this sense, the presence of 1p/19q codeletion along with an IDH mutation is a key diagnostic and prognostic factor since IDH-mutant and 1p/19q-codeleted gliomas demonstrate increased sensitivity to chemotherapy, which gives them a much more favorable prognosis than tumors that do not have these mutations. IDH mutations occur in only 6% of the pediatric population, but the percentage increases significantly with age, reaching more than 16% among adolescents and young adults. The authors describe in detail the immunophenotype, DNA methylation profiling, proliferation pattern, and vasculature and other molecular characteristics of these types of gliomas, which determine the diagnostic criteria for IDH-mutant astrocytoma classification by the WHO (World Health Organization). The authors also provide updated data on preclinical studies involving various IDH inhibitors, vaccine therapies, and poly(adenosine diphosphate ribose) polymerase (PARP) inhibitors (Valvi et al.). Regarding treatment, it is known that there is a window of time during which IDH-mutant gliomas show little progression, but once malignant transformation occurs, the tumor rapidly becomes aggressive. This is particularly important in the adolescent and young adult population, where there is a high likelihood of success if treatment is initiated early.

Dr. Huang and colleagues (Department of Neurosurgery, The People's Hospital of Lichuan City, Lichuan, Hubei, China) focus their bibliometric analysis on advances in proton therapy for glioma (Liu et al.). The United States—specifically Massachusetts General Hospital—has led research in this therapy over the past 30 years. Proton therapy is a promising approach to radiation therapy, as it targets the tumor while exposing the surrounding healthy tissue to very little radiation. This offers clear advantages in terms of toxicity profiles compared to conventional photon-based radiation therapy, which is more established in clinical practice. The literature review shows a clear increase in publications related to proton therapy over time, with 2024 being the most productive year. The published articles aim to improve the efficacy of this therapy by reducing its impact on neurological function, enhancing its safety, and improving the assessment of the risk of radiation-induced necrosis. The studies place particular emphasis on the advances made in pediatric oncology with this therapy. Given the results obtained so far, the authors propose continuing to optimize proton therapy to maximize its benefits, including lower costs and reduced uncertainties (Liu et al.).

Literature reviews address various aspects of glioma, including its impact on the gut microbiota. In this regard, Dr. Zhao and his team (Department of Neurosurgery, Northwest University First Hospital, Xi'an, Shaanxi, China) point out that the countries that conduct the most research on this topic are China and the United States, particularly the MD Anderson Cancer Center at the University of Texas in the United States and Central South University in China (Chen et al.). In addition to its role in immune function and inflammation, the gut microbiota may also affect the efficacy of immunotherapy. In this regard, it has been suggested that modulating the gut microbiota may influence the response of gliomas to new therapies, such that the composition of patients' microbiota may improve their prognosis. Although this approach has been the subject of increased research recently, there are no systematic reviews to help clarify the latest research findings, methods, and data. This study shows that, once again, 2024 is the year with the highest number of publications. Over the past two decades, there has been an increase in the number of studies addressing the importance of the gut-brain axis in central nervous system disorders, and it has been reported that the gut microbiota is specifically associated with the pathogenesis of glioma and that this relationship is bidirectional, so that changes in the gut microbiota can influence brain neurotransmitters or the permeability of the blood-brain barrier. Although several countries have begun to explore the management of the microbiome and its relationship to glioma therapies, the authors emphasize that greater collaboration among different countries is needed to make further progress in this area, as has been done, for example, with the predictive value of microbiota markers evaluated by the GLIOTRAIN consortium (comprising eight European countries and Australia). They also suggest several strategies related to dietary interventions, including probiotics, that are currently being studied (Chen et al.).

A systematic review (according to PRISMA 2020 guidelines) on the rebound regrowth phenomenon in patients with pediatric low-grade gliomas treated with mitogen-activated protein kinase (MAPK) inhibitors has been performed by Dr. Styczewska and co-workers (Department of Pediatrics, Hematology and Oncology, Medical University of Gdansk, Gdansk, Poland) (Styczewska et al.). Tumor regrowth following MAPK inhibitor discontinuation is known as rebound regrowth (radiological progression within 6 months after treatment cessation) which appears clinically and biologically different from acquired resistance to MAPK inhibitors or classic off-therapy progression (Styczewska et al.). Rebound regrowth events were most frequent in tumors harboring the BRAF V600E variant and most patients responded to MAPK inhibitor rechallenge; this suggests preserved drug sensitivity (Styczewska et al.). The authors stated that dose-tapering strategies, structured post-discontinuation surveillance, prospective evaluation of treatment duration, and standardized definitions are required to optimize MAPK inhibitor discontinuation and long-term disease management in patients with symptomatic or progressive pediatric low-grade gliomas (Styczewska et al.). This systematic review highlights the importance of having in-depth knowledge of the predictors, timing, and incidence of rebound regrowth events.

Dr. Ren and collaborators (Department of Radiation Oncology, General Hospital of Northern Theater Command, Liaoning, China) have reported a case report of a 50-year-old male diagnosed with right temporal glioblastoma (WHO grade IV) who underwent surgery and in whom the Stupp protocol was applied (radiotherapy + temozolomide) (Ren et al.). Later, a second tumor appeared (small-cell lung cancer: cT3N2M0, stage IIIB) and then the patient received continued cranial radiotherapy with temozolomide alongside etoposide plus platinum chemotherapy and subsequent palliative thoracic radiotherapy for the lung tumor (Ren et al.). This procedure was well-tolerated (no grade ≥ 3 adverse events), resulting in stable intracranial disease and partial response of the pulmonary lesion. The patient experienced an overall survival of 58 months from initial glioblastoma diagnosis, significantly exceeding the typical prognosis for either tumor alone (Ren et al.). The co-occurrence of glioblastoma and small cell lung cancer is very rare, but this case report suggests that a concurrent chemoradiotherapy strategy may accomplish dual tumor control, conferring survival benefits in patients suffering from both cancer types.

The study carried out by Dr. Maccari and collaborators (Department of Surgery, Oncology and Gastroenterology, University of Padova, Padova, Italy) retrospectively evaluated outcomes of targeted and systemic therapies for recurrent adult ependymomas (single-center analysis) (Maccari et al.). Patients suffering from intracranial or spinal ependymoma and receiving at least one systemic line were studied (Maccari et al.). Sequential systemic therapy, up to five lines, was feasible in selected cases (Maccari et al.). Tumor response was assessed according to the RANO criteria. Treatments were in general well-tolerated, with limited grade 3 toxicities, and the primary endpoints were progression-free survival and disease control rate, whereas secondary endpoints were overall survival from diagnosis and overall survival from the start of the first line treatment (Maccari et al.). The authors stated that this descriptive real-world analysis demonstrated that temozolomide was feasible and associated with disease stabilization in selected patients; that bevacizumab alone achieved a disease control rate of 33% with one case of prolonged stabilization; that the combination of temozolomide and lapatinib had a limited benefit; that bevacizumab plus fotemustine yielded a progression-free survival of 14.1 months, and that bevacizumab-based combinations showed signals of clinical activity in recurrent adult ependymomas (Maccari et al.). This study suggests that real-world registries are crucial for optimizing treatment sequencing and improving outcomes in ependymomas.

The group headed by Dr. Zhang (National Cancer Center, Cancer Hospital Shenzhen Hospital, Shenzhen, China) conducted a single-center retrospective study on the efficacy and safety of postoperative hypofractionated radiotherapy combined with temozolomide in patients with newly diagnosed glioblastoma with poor prognosis (rapid early progression, recursive partitioning analysis class IV–VI) (Fu et al.). Hypofractionated radiation therapy regimens, which mainly alter tumor kinetic dynamics, allow for shorter treatment schedules and deliver higher per-fraction doses (Fu et al.). Most of the treated patients showed unfavorable tumor characteristics; the median progression-free survival was 8.4 months, and the median overall survival was 17.7 months (Fu et al.). Acute grade ≥ 3 toxicities occurred in 16.7% of patients, all managed conservatively, and no radiation necrosis was observed. Hypofractionated radiotherapy-temozolomide treatment is a promising therapeutic alternative because this combination showed encouraging survival outcomes and manageable toxicity in glioblastoma patients with poor prognosis who are unsuitable for standard therapies.

Continuing with retrospective studies, Dr. Marques-Torrejon's team (Department of Medicine, Jaume I University of Castellon, Castellón de la Plana, Spain) has analyzed the clinical data of 57 glioblastoma patients treated at the General University Hospital of Castellón, Spain (Gutiérrez-Arroyo et al.). This is an exploratory study (excluding patients with IDH-mutant astrocytomas) that assesses the impact of several variables on overall survival and progression-free survival in particular: clinical, tumor-related, treatment-related, lifestyle, and genetic factors. Of all the variables studied, the authors found a strong correlation between survival and the extent of resection, and particularly with the tumor's proximity to the lateral ventricles (ventricular proximity was independent of the surgical approach). The authors found that the closer the tumor is to the ventricular system, the shorter the survival period, this proximity is therefore a crucial prognostic factor. Other factors such as sex, age, treatments, tumor volume, or genetic mutations showed no significant influence on survival. The authors propose that cerebrospinal fluid or the subventricular zone's (SVZ) trophic niche may contribute to tumor progression by promoting initial tumorigenesis and facilitating recurrence following treatment. The results highlight the importance of a multidisciplinary approach to designing personalized treatments, with particular attention to the tumor's proximity to the lateral ventricle, in order to improve patient prognosis (Gutiérrez-Arroyo et al.).

The involvement of the SVZ and the impact of radiation on this area on the survival of a homogeneous cohort of glioblastoma-treated patients are addressed in an exploratory, prospective study conducted by Dr. Pillai and colleagues (Gujarat Cancer and Research Institute, Ahmedabad, India) (Pillai et al.). The results confirm that tumor contact with the SVZ is a marker of poor prognosis in patients with glioblastoma treated uniformly with maximal safe resection and temozolomide-based chemoradiation using IMRT/VMAT. The authors have used standardized SVZ contouring and detailed dosimetric analysis of ipsilateral, contralateral, and bilateral SVZ dose parameters, as well as correlation of SVZ contact and dose thresholds with progression-free and overall survival. Higher incidental irradiation of the ipsilateral SVZ during postoperative radiotherapy may be associated with improved survival. This reinforces the hypothesis that SVZ irradiation may suppress stem-cell-mediated tumor regrowth, although the authors described a limitation of the study, namely the small sample size (28 patients). They expect that ongoing and recently completed clinical trials, including NCT01478854, NCT02039778, and NCT02177578, will provide more definitive evidence regarding the therapeutic value of SVZ modulation in glioblastoma and may ultimately inform future treatment paradigms for this highly aggressive disease (Pillai et al.).

The articles published in the second volume of this Research Topic range from molecular aspects (Joshi et al.; Yan et al.; McDaid et al.) to patient-based studies (Styczewska et al.; Ren et al.; Maccari et al.; Fu et al.; Gutiérrez-Arroyo et al.; Pillai et al.), including review articles (Ribeiro et al.; Valvi et al.; Liu et al.; Chen et al.) that seek to integrate various approaches to glioma treatment. The studies highlight the enormous complexity of these tumors, considering very specific tumor mutations or their relationship with the gut microbiota, which may seem far removed from the tumor site. Although such a diverse range of approaches can be overwhelming due to the many factors that must be considered, it is encouraging to see that the problem is being addressed in a multidisciplinary manner. All these studies will undoubtedly contribute to a better understanding of glioma, which will make it possible to develop appropriate and personalized treatments that will benefit patients.

